# “‘Academic’ is a dirty word”: Intended impact pathways of an emerging academic health centre in tropical regional Australia

**DOI:** 10.1002/hpm.2681

**Published:** 2018-10-12

**Authors:** Alexandra Edelman, Judy Taylor, Pavel V. Ovseiko, Stephanie M. Topp

**Affiliations:** ^1^ College of Public Health, Medical and Veterinary Sciences James Cook University Townsville Queensland Australia; ^2^ College of Medicine and Dentistry, Division of Tropical Health and Medicine James Cook University Townsville Queensland Australia; ^3^ Radcliffe Department of Medicine, Medical Sciences Division, University of Oxford John Radcliffe Hospital Oxford UK

**Keywords:** academic health centre, Australia, health systems, regional, research translation

## Abstract

**Background:**

The Tropical Australian Academic Health Centre (TAAHC) is being established in northern Queensland across a vast rural geography. The study aim is to identify intended impact pathways and beneficiaries of TAAHC as well as experienced and anticipated challenges.

**Methodology:**

The study is an empirical case study nested within a comparative multi‐case study on academic health centres (AHCs). Data were collected from documents, observation, and interviews with 24 health system and university stakeholders. Intended impact pathways were identified abductively from analysis of aspirations and challenges.

**Results:**

Aspirations of TAAHC reflect an ultimate aim to improve the health of the northern Queensland population. Challenges were trust and communication, understanding value and return on investment, health system receptiveness to building a research culture, prioritising and influencing the research agenda, and structure of the health system.

**Discussion:**

The study identifies three interdependent transitions that comprise the main intended impact pathway in TAAHC. Stakeholders expected TAAHC to effect health systems change and improvement rather than drive discovery‐oriented academic research associated with AHCs elsewhere.

**Conclusion:**

The findings contribute to the empirical evidence base on the role of AHCs internationally and to ongoing initiatives to establish and resource AHCs in Australia.

## 1 INTRODUCTION

Academic health centres (AHCs) have been established in multiple countries across the world with the mandate to advance research‐informed health service delivery and workforce education and training.^1^ The establishment of structures with the similar AHC mandate in Australia commenced within the recent decade, drawing from international experience and propelled by federal‐level and state‐level commitments to foster “translational” research.^2^ Against a backdrop of major policy reports recommending the establishment of AHC structures nation‐wide,^3^, ^4^ some health system and university leaders moved to establish AHCs around existing health precincts and collaborations, and a formal “designation” process, led by the National Health and Medical Research Council (NHMRC), commenced in 2014.^5^ AHCs are termed by the NHMRC “Advanced Health Research and Translation Centres” (AHRTCs). A feature of the Australian approach was an early policy recognition of the potential for the new AHC structures to have a focus on improving health outside of urban centres,^3^ and exemplifying this focus, another regional category was added in the second NHMRC designation round termed “Centres for Innovation in Regional Health” (CIRHs). Unlike AHC models internationally, the word “academic” was not used in this nomenclature reflecting the NHMRC's intention that the initiatives be led by “Australia's health care system itself” rather than universities.^6^ Nonetheless, some AHRTCs and CIRHs have sought to retain the term “academic” in their titles, sometimes combining the international and NHMRC terminology.^7^, ^8^, ^9^

Designation of AHCs in Australia mirrors the processes undertaken in the United Kingdom^10^ and involves a call for submissions and assessment by an international panel of experts. Criteria for designation include evidence of health and medical research excellence and translation pathways, leadership in research‐based and evidence‐based clinical care and health professional education, and collaboration among partners.^5^ Like in the United Kingdom, successful designation in Australia does not include funding but serves as formal recognition of clusters of excellence in joined‐up service delivery, research, and health professional education. While some AHCs in their early stages of establishment received start‐up funding from state governments, federal funding for designated centres soon followed the designation process, with the 2016/17 and subsequent federal budgets committing funds from the new Medical Research Future Fund to enable their translationally focussed research activity and operations.^11^ A third designation round for AHRTCs, and a second for CIRHs, is expected to be announced in 2018.

Despite the proliferation of AHC structures across the world, the literature on AHCs is dominated by opinion papers focussed on the North American context, with little empirical or theory‐driven inquiry.^1^ In addition, despite the apparent high expectations of their contribution, the literature betrays a degree of uncertainty among experts about what AHCs ultimately exist to achieve and to whom they deliver benefit.^12^ In the Australian context, a need has been identified for research that examines the barriers to the successful establishment of AHC structures and what mechanisms might enable their success.^2^ In this paper, we address some of these gaps by presenting a case study of a regional Australian AHC which is being established in northern Queensland. The aim of the study is to empirically identify intended impact pathways and beneficiaries of the AHC initiative, and the key challenges. We are specifically interested in why the AHC is being established and for whom; how the AHC is expected to achieve its aims; and what the experienced and anticipated barriers are to the AHC's “success.”

## 2 STUDY SETTING AND CONTEXT

**Figure 1:**
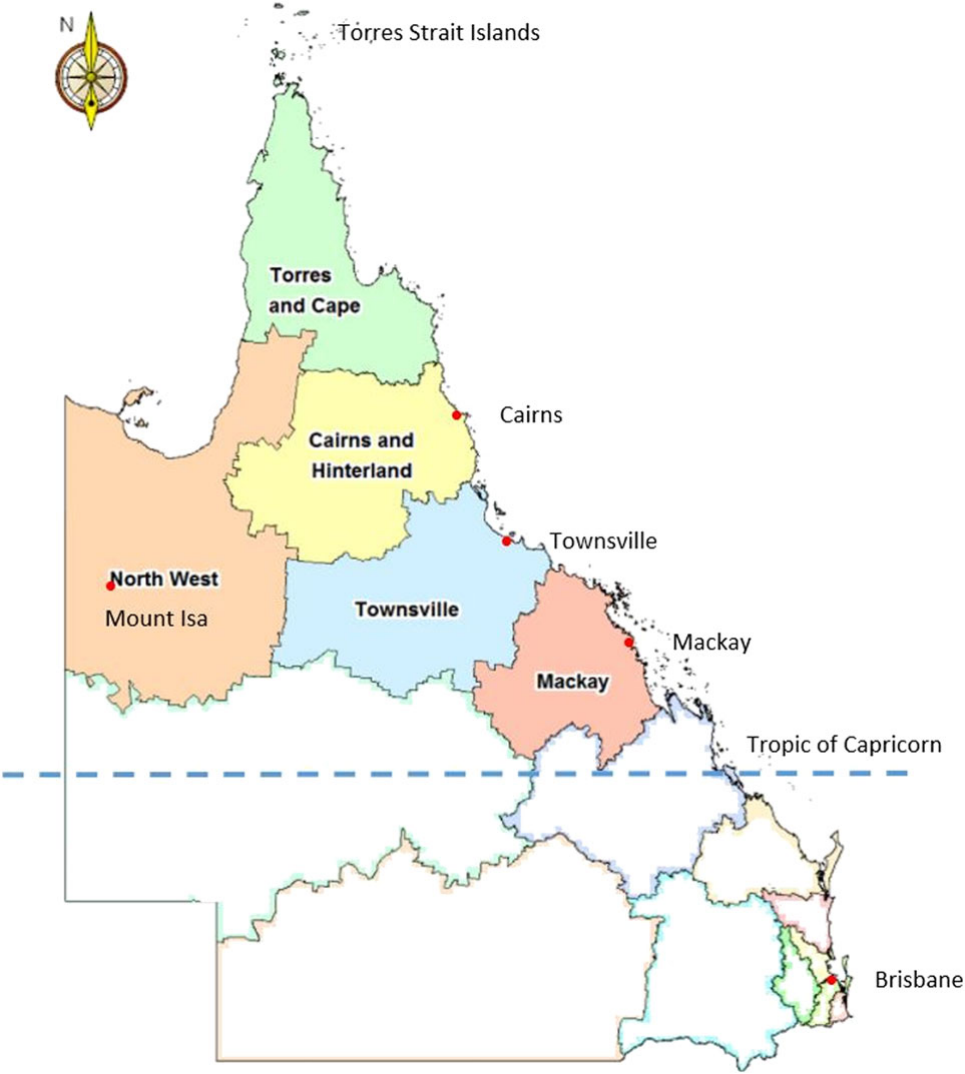
Hospital and health service jurisdictions within the State of Queensland, Australia. The shaded jurisdictions above the Tropic of Capricorn are among the founding TAAHC member organisations and collectively indicate the geographic boundary of the TAAHC initiative at its inception

The Tropical Australian Academic Health Centre (TAAHC) is undergoing establishment in northern Queensland (Figure 1). Across a vast geography of around 850 000 square kilometres—an area more than three and a half times the size of the United Kingdom—TAAHC's stated aims are to improve service delivery, health workforce education and training, and tropical health and medical research targeting the health needs of a highly dispersed population.^13^ The region's population of approximately 750 000 people is largely clustered within the regional cities of Cairns, Townsville, and Mackay, but a large proportion live in small towns and communities located in areas classified as rural and remote.^14^, ^15^ Life expectancy in the rural and remote areas is lower than in the regional centres and in Queensland as a whole, with these areas experiencing substantially higher rates of premature and avoidable mortality and potentially preventable hospitalisations.^14^, ^15^ Improving the health of Aboriginal and Torres Strait Islander populations living in the region is a major priority of the region's health care as well as academic organisations.^14^, ^15^ Northern Queensland's proximity to South East Asia and the West Pacific also contributes to a focus in health research on tropical health and multi‐morbidities.^16^

**Figure 2:**
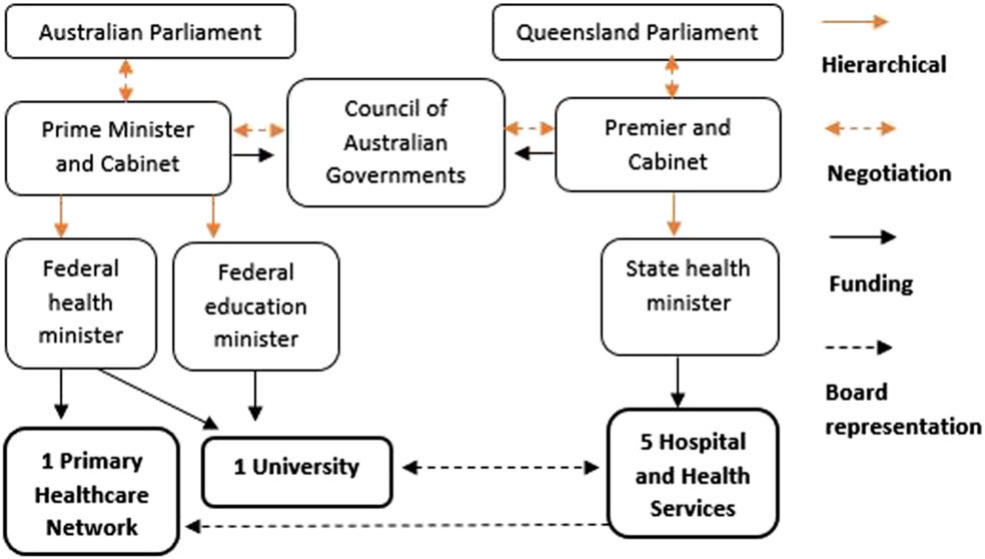
Structural relationships between the TAAHC partner organisations demonstrating the “unlinked partners” model of academic‐clinical relationships in Australia. Adapted from the “Organization of the Health System Australia,” in *International Profiles of Health Systems,* the Commonwealth Fund, 2017

The founding partners of TAAHC comprise five Hospital and Health Services (HHSs), the northern Queensland Primary Health Network and James Cook University which includes the Australian Institute of Tropical Health and Medicine. HHSs are statutory agencies that deliver a range of services across the health care continuum and are funded by the Queensland Government through service agreements negotiated between the health department and the HHS governing boards. Primary Health Networks are funded by the Commonwealth Department of Health to procure health and medical care services and improve coordination of care and operate in the region as planning and commissioning agencies. The academic‐clinical relationships within TAAHC, similar to relationships between partnering organisations within AHCs in other jurisdictions across Australia, represent the “unlinked partners” model wherein the university and the collaborating health service organisations employ separate governance and reporting arrangements with no overarching executive authority across the patient care, research, and education missions.^17^, ^18^, ^19^ Figure 2 shows the structural separation of the component organisations in TAAHC and the nature of existing and emerging governance relationships.

The respective responsibilities of health services in Australia are set out in the National Health Reform Agreement (2011) developed through the Council of Australian Governments, which is the peak intergovernmental forum that manages matters of national significance and matters requiring coordinated government action, such as relating to health care and higher education. Australian public universities, many of which are established through state and territory legislation, are predominantly funded federally. Between the TAAHC organisations, various local‐level governance arrangements exist to support collaboration and shared decision‐making, including cross‐organisational representation on governing boards and committees, shared clinical/academic appointments, and memoranda of understanding relating to collaboration in clinical training of health professional students and joint precinct management.

In 2017, the TAAHC partners commenced a process of formalising the collaboration through creating a company limited by guarantee governed by a constitution and members' agreement. At the time of data collection, a Steering Committee comprising representatives from each member organisation was meeting periodically either in person or by virtual meeting platform, and a number of subcommittees had also been convened for specific activities. James Cook University also provided in‐kind “back office” support and administered a small pool of resources to support establishment activity, which comprised negotiated financial contributions from each of the member organisations. A memorandum of understanding was signed by the partners in 2016 to indicate shared commitment to progress establishment of TAAHC. The new TAAHC constitution provides for establishment of a board comprising two directors from each founding member of TAAHC and a tiered system for establishing membership fees. The constitution also provides for resolutions to be passed by a majority of the votes cast by directors present and entitled to vote. Even with the creation of the TAAHC company, there remains no single executive authority over the member organisations' patient care, research, and education functions.

## 3 METHODS

### 3.1 Study design

The study design adopts a social science approach to case study research which sees the case study as a form of empirical inquiry that seeks to understand complex social phenomena within real world settings.^20^, ^21^ The TAAHC case study is nested within a larger multi‐case study project which is exploring the equity‐related aims and activity of four unique AHCs in two countries: Australia and the United Kingdom. TAAHC was selected for its self‐identification as an AHC in Australia with a documented focus on improving health for rural, remote, and Aboriginal and Torres Strait Islander populations within its geographic vicinity.

### 3.2 Data collection

The TAAHC case study involved data collection between October 2017 and March 2018 from three sources: semi‐structured interviews with core stakeholders; observation of AHC activity within the AHC settings over multiple days (recorded in research memos); and documentation. Within the TAAHC and other case studies, the researchers' intention in using multiple data sources and collection methods was to increase the rigour of data analysis and to enable corroboration of findings though triangulation of the data.^20^

#### 3.2.1 Interviews

Selection of interviewees aimed to achieve representation of different perspectives including professional backgrounds, level of seniority, and gender, from within an identified “core stakeholder” group, defined as individuals in positions to drive, shape, and implement the AHC direction, structures, and key activities. A total of 25 interviews were conducted involving 24 interviewees (see Table 1) and representing all TAAHC member organisations. An interview guide was developed, pilot‐tested with a health system executive familiar with the TAAHC initiative, refined and used in all interviews by the lead author. All interviewees were given an information sheet about the project and intent of the interview, and consented to their interview data being used. All but three interviews were recorded and transcribed; for those that were not recorded at the interviewees' request, the interviewer took handwritten notes during the interview. A process of member‐checking was undertaken involving sending interviewees summaries of the results and inviting feedback by email or phone.

**Table 1:** Data collection methods including interviewee characteristics

Interviews (*n* = 24). Average Duration: 35 min (Range: 10 to 75 min). Method: 21 in Person; 3 Phone	
*Role type* a *and number of interviewees*	*Relationship to the TAAHC initiative*
Health system executives (HSE) (*n =* 13)	Direct involvement in TAAHC establishment and decision‐making. Many are members of the TAAHC governing body.
University executives (UE) (*n* = 5)	Direct involvement in TAAHC establishment and decision‐making. Some are members of the TAAHC governing body.
Clinical academics (CA) (non‐executive level) (*n* = 4)	Hold clinical roles within TAAHC health service organisations and participate in/lead clinical research activity at the university. Central to the TAAHC research and translation agenda.
Non‐clinical academics (NCA) (non‐executive level) (*n* = 2)	Non‐clinicians involved in research and teaching at the health system/university interface. Central to the TAAHC research and education agenda.

Observation Memos (*n* = 5)	
Researcher observations on space, actors, activity, and goals. Features and activities observed included co‐location or distance between executive offices of TAAHC member organisations, nature and mode of interactions between the partnering organisations, executive operating environments, and visible organisational goals.	

Documentation (*n* = 8)
TAAHC review report, 2017; two TAAHC steering committee meeting minutes (December 2015; and May 2016); TAAHC NHMRC CIRH submission and cover letter, 2016; TAAHC business case, 2017; TAAHC research development strategy, 2017; TAAHC presentation, 2016; TAAHC memorandum of understanding (MOU) 2016.

a Where interviewees held multiple roles across the different organisations (for example, held roles as a university academic and a health service board member simultaneously), they were allocated to the role type that best reflected their current or likely interaction with the TAAHC initiative.

#### 3.2.2 Observation

The lead researcher's physical attendance at each of the TAAHC member facilities, equating to 2 to 3 days in each of the four main executive office locations of the TAAHC member organisations (Cairns, Mackay, Mt Isa, and Townsville), enabled unstructured non‐participant observation with reference to four of Spradley's (1980) nine dimensions of observation: space (physical places), actors (the people involved), activity (a set of acts that people do), and goals (the things people are trying to accomplish).^22^ Researcher reflections on these observations were captured in five written memos. Attendance as an observer at formal meetings of TAAHC steering committee was requested of the TAAHC Chair, but at the time of data collection few meetings were taking place, and the request was declined. Documentation relating to member organisations' strategic objectives (eg, annual reports, study prospectuses, and marketing material) were accessed as part of the observation of organisational goals.

#### 3.2.3 Documentation

Eight TAAHC‐specific documents were accessed and analysed with the approval of the TAAHC Chair.

### 3.3 Data analysis

Interviews were transcribed verbatim (or typed in the case of handwritten notes), read, and coded inductively by the lead researcher into descriptive codes and categories using NVivo software. These codes and the coding process were subsequently discussed and refined by co‐researchers. The categories were then aggregated into themes corresponding to the study's overarching research questions. Researcher memos from the observation, and documents, were also coded in NVivo and were used to triangulate the findings emergent from interviews. A logic model was developed to describe aims and expectations of stakeholders and intended impacts of TAAHC, which constituted a “coding tree” reflecting the data on aspirations. Logic models can be used in case study research as an analytic technique to help explain the ultimate intended outcome of a program.^20^ Because of the emphasis of TAAHC on fostering translational research, the logic model structure used within the Framework to Assess the Impact from Translational health research^23^ was selected and adapted for this purpose. To describe impact aspirations, the Canadian Association of Health Sciences' five categories to track research impact^24^ were used to classify interviewees' perceptions of potential indicators of the success of TAAHC. Intended impact pathways were identified abductively and linked to thematic synthesis of data on barriers and challenges.

### 3.4 Researcher reflexivity

As some members of the research team were employed by James Cook University, and one was involved in the establishment of TAAHC prior to the commencement of the case study, a process of researcher reflexivity was employed to minimise bias. This involved the researchers being alert to avoid projecting their own experience in data collection and analysis,^25^ as well as use of open‐ended processes of inquiry, immersion in the data, seeking of complementary and divergent opinions, and use of memos to document assumptions.^26^ Although this project represented the first qualitative research project undertaken by the lead researcher, all other members of the research team had extensive qualitative research experience as senior health systems researchers, increasing the internal validity of the study. One member of the research team had previously conducted research on AHCs in the United Kingdom and was involved in the establishment of an AHC in Oxford.

## 4 RESULTS

The findings on aspirations and challenges are supported by verbatim quotes from interviews, memos, and documentation. Each quotation is identified using an acronym relating to profession type (health system executive = “HSE”; university executive = “UE”; clinical academic = “CA”; non‐clinical academic = “NCA”) and a number corresponding to their random order in a list of interviewees in each professional grouping.

### 4.1 TAAHC aspirations

**Figure 3:**
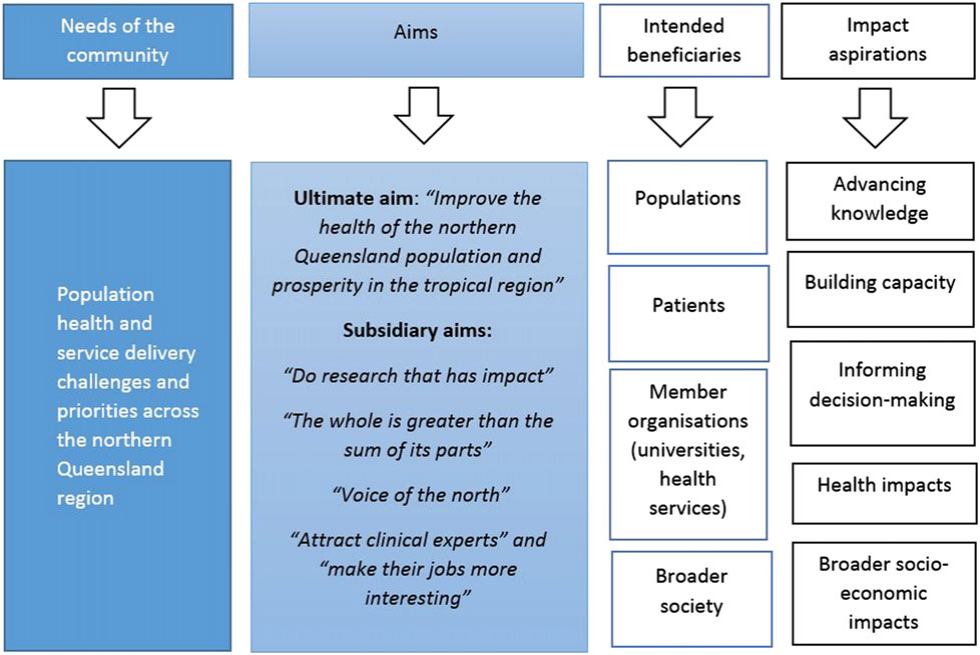
Logic model showing perceptions of the aims, intended beneficiaries, and aspirational impacts of TAAHC. Impact aspirations are described using the Canadian Academy of Health Sciences impact categories

Figure 3 presents a logic model showing “needs of the community” as a starting point for the TAAHC program, as well as TAAHC aims, intended beneficiaries, and impact categories, adapted from Framework to Assess the Impact from Translational health research.^23^ Each heading of the logic model represents a theme.

#### 4.1.1 Needs of the community

Multiple health systems challenges, needs, and priorities were identified by interviewees either as drivers of the establishment of TAAHC, or at least as key contextual conditions influencing the focus and development of TAAHC. Interviewees and documentation emphasised population health and service delivery challenges such as managing high rates of chronic disease and infectious disease outbreaks and incursion risks, as well as management challenges such as meeting growing demand for health services with finite budgets. High levels of socioeconomic disadvantage in the community were described, and a need was identified to enhance chronic disease risk prevention particularly for vulnerable groups. Some interviewees saw a need for a northern‐Queensland‐wide or “zonal” perspective in health service planning to improve integration and coordination between primary and secondary care. Referencing a small remote town 800 km inland as compared with a larger regional coastal city with a teaching hospital, one interviewee described this as thinking about how “a person in Cloncurry or on a [remote] station can have an equivalent outcome should they get a stroke, as someone in Townsville” (UE1).

#### 4.1.2 Aims and intended beneficiaries

##### “Improve the health of the northern Queensland population and prosperity in the tropical region” (TAAHC MOU, 2016).

Nearly all interviewees saw the concept of establishing an AHC as ultimately aimed at improving the health of people living in northern Queensland; this aim was also described within the TAAHC MOU as a vision to “improve the health of the northern Queensland population” (2016). Patients were also identified—mainly by health system executives—as the intended beneficiaries of TAAHC, with one suggesting that improving “patient care” is central to its rationale (HSE3). Many interviewees saw TAAHC as a mechanism to improve the region's health system; one health system executive described it as representing the next stage of the system's “maturation” from a system “lurching from service delivery crisis to crisis” around 15 years ago to one currently able to offer “safe and reliable service delivery” underpinned by a self‐sustaining health workforce (HSE5). Another health system executive, however, saw TAAHC as more concerned with developing a “unique selling point” and research profile than with addressing local health systems issues (HSE9).

Beyond northern Queensland, TAAHC strategic documentation articulated a vision to improve “prosperity in the tropical region,” alluding to an aspiration to extend its influence to international populations, which some interviewees understood to involve a focus on the Asia Pacific region (UE4; HSE10). The word “prosperity” also indicated a focus within TAAHC on wealth generation which was also evident in documented objectives relating to “developing new industries in life sciences innovation” (TAAHC MOU, 2016). One interviewee similarly saw TAAHC as positioned to “see the development of health industries that will service populations near and far” (HSE12).

##### “Do research that has impact” (UE3)

Building research capacity was understood to be the central, immediate focus of TAAHC, and research was seen by interviewees and described in documentation as the key vehicle for health systems improvement. Interviewees who did not articulate this understanding had, at the time of interview, little direct interaction with the TAAHC initiative or were only introduced to it recently; they nonetheless articulated an expectation that fostering research within health service settings was an important way to improve health systems and outcomes:

“There are opportunities for research to look at primary health care [in our region], including evidence based models of care and interaction between primary health care and acute care...Research should have impact on the whole of the system.” ( HSE2)

Multiple interviewees pointed to a range of existing initiatives that aimed to build this research capacity (HSE2; NCA2; HSE3; UE3; NCA1), some of which involved health service‐university collaboration, but none reflected all of the organisations involved in TAAHC. The words “impact,” “translation,” “applied,” and “health services” were used in documentation and by interviewees to describe the *type* of research that stakeholders saw as needed and as potentially driven by TAAHC:

“TAAHC is [about] having [a] critical mass to be able to do research that has impact that is able to make a difference.” ( UE2)

A health system executive saw the research focus in TAAHC as potentially giving clinicians space and permission to ask “challenging” questions about their practice, to make them “a better consumer of the delivery of health care services” (HSE5). Another interviewee similarly saw the establishment of TAAHC as a “practical” initiative aimed at putting an “evidence base behind solutions for some outcomes” within the health system (UE5). Reflecting on the national context, one interviewee saw the development of AHCs in Australia as a product of broader debates about growing Australia's “translational research” capacity in contrast to “basic research” (UE2).

##### “The whole is greater than the sum of its parts” (HSE5)

Key to growing the type of research that was seen to be needed was the notion that it had to be done through a collaborative structure involving multiple health service delivery and academic institutions. Documentation referred to TAAHC growing the participating organisations' “collective capability in tropical health and medical research, health care and workforce development” (TAAHC Business Case 2017). One interviewee emphasised the need to overcome organisational boundaries in order to improve the quality and impact of research output:

“The problem with universities is they're not so closely engaged in a clinical setting, and the problem with hospitals is they want to do research but they don't have the academic links to provide the quality of research. So what we end up with is potentially unethically investing in a lot of research that isn't well designed, that engages patients in research [but] that provides outcomes that aren't that valuable.” ( CA4)

The TAAHC structure was understood by interviewees to be the only region‐wide formal collaboration mechanism between all of the organisations involved in TAAHC. As such, enhanced inter‐institutional collaboration was seen by some as beneficial beyond just enabling increased, better quality and “impactful” research effort; it was seen as an enabler of region‐wide strategic planning and “coordination” of different types of activity:

“TAAHC is a systems approach that brings together disparate models and ways of thinking about it [health care] … the overriding thing is that you synthesise something greater by bringing all these different bits together.” ( UE4)

Tropical Australian Academic Health Centre was similarly described by one interviewee as embodying the notion that “the whole is greater than the sum of its parts” (HSE5). To illustrate the possibilities, the TAAHC Review Report suggested that TAAHC oversee activity to “coordinate approaches to building and disseminating evidence‐based practice across the TAAHC region” in areas such as disaster response and public health planning (2017).

##### “Voice of the north” (HSE13)

Tropical Australian Academic Health Centre was also seen as a strategy to enhance access to government and other funding sources for translational research, and to increase the status and reputation of the partnering organisations and the region as a whole. One interviewee described TAAHC as potentially enabling a positive feedback loop between increased research capacity, the TAAHC brand, and access to funding:

“If you're a success story, then you attract success, you bring people who want to work with you, you bring donors, you bring government programs, you know it's the honey pot.” ( UE3)

Another interviewee described the potential for TAAHC to grow “the voice of the north” in Queensland, which could in turn lead to a redistribution of resources from large metropolitan‐based HHSs to rural parts of the state:

“Here's where TAAHC can help, because everything is Brisbane‐centric, and the likes of the Metro South and North [HHSs] – they're big players and they're vying for a piece of the [funding] pie too … I see the purpose of TAAHC being able to … get the ‘north’ out there and to get the research activity and our uniqueness out there.” ( HSE13)

Some interviewees and documents emphasised TAAHC's marketability as “rural,” “remote,” and “tropical” (CA2; TAAHC CIRH submission, 2016); differentiators from other urban parts of Australia with longer histories of access to research resources.

##### “Attract clinical experts” (CA2) and “make their jobs more interesting” (CA1)

Attracting and retaining a competent and capable health workforce were discussed particularly by interviewees from within the health system as a key aim of TAAHC, and this was closely linked to the potential for TAAHC to grow the region's capacity in translational research:

“In order to attract … clinical experts up here, we need to be able to offer them really robust research support and a good track record in research.” ( CA2)

Increasing the reputation of the region's health system was also seen as a strategy to attract talented staff, with the existence of TAAHC potentially differentiating the health service partners from “just another hospital that delivers patient care” (HSE13). TAAHC was also seen as a vehicle for increasing the skills and capabilities of existing staff, and in so doing, “mak [ing] their jobs more interesting, reducing professional isolation and getting people more involved in reflective practice and improving the quality of services and outcome” (CA1).

#### 4.1.3 Expected “impacts” of TAAHC

Although no set of agreed TAAHC performance metrics had been developed at the time of data collection, interviewees described their expectations of the types of metrics that could be used to measure its “success.” We used the Canadian Academy of Health Sciences (CAHS) “systems approach” to capturing research impact to classify these expectations into five main categories: advancing knowledge, building capacity, informing decision‐making, health impacts, and broader socio‐economic impacts.^24^

##### Advancing knowledge

Traditional academic metrics, such as “grant income” (CA2), “contribution to the body of knowledge by publication” (HSE11), and Excellence in Research for Australia league table rankings for the university partner (UE1), were identified by interviewees across all professional role groupings as necessary indicators to track the progress of TAAHC. Interviewees recommended these only as adjuncts to a suite of other more translationally‐focussed indicators, suggesting that they were not by themselves sufficient to gauge TAAHC's success.

##### Building capacity

Within the CAHS framework, “building capacity” incorporates metrics on enabling factors such as personnel, funding, infrastructure, and less tangible factors such as receptor or absorptive capacity. In this area, some interviewees emphasised tangible capacity building indicators such as “increased enrolments in higher education” (UE1), increased clinician recruitment (HSE11), numbers of clinician researchers trained in postgraduate research supervision (CA2), and inclusion of other organisations within the TAAHC collaboration (HSE9). More often, however, interviewees emphasised less tangible indicators including clinicians' sense of being supported to do research and workplace satisfaction levels (HSE8), recognition at different levels that research has value in health care (UE2), and brand recognition of the TAAHC entity: “when it rolls off peoples' tongue” (UE3). One interviewee reflected that TAAHC would be successful when staff working in the region's health system had a sense of pride in their location, such that “… the staff that work for us see themselves as equal to any other service provider in Australia.” (HSE5).

Other less tangible metrics related to collaboration between the parties to TAAHC; these were discussed mostly by health system executives, some of whom saw “success” looking like: “… a robust governance process [with] the partners all working on a collaborative vision” (HSE1); “visibility of genuine partnership … between the health service and the other health service providers in the region” (HSE8); and “all the players in the north singing the same tune, on the same page” (HSE13). Another health system executive sought to emphasise the importance of collaboration as an indicator of success:

“I can't stress enough how important it is to have an environment where researchers and clinicians can work closely together. That in itself is improving outcomes.” ( HSE4)

##### Informing decision‐making

In the CAHS framework, informing decision‐making metrics represent the pathways from research to health and other social outcomes, and include health‐related, research, industry, and general public, decision‐making. In this category, multiple interviewees discussed the importance of developing metrics to measure pathways from research to health service improvement, with one noting the potential for:

“… longitudinal measures of improvement in health outcome … [to be] directly attributable back to research that were generated by or translated by the influence of TAAHC. So you're seeing a difference. And that difference can be attributed to the strategy development of TAAHC.” ( HSE5)

A clinical academic similarly suggested the possibility of developing metrics around “measuring translation of research into actual service provision” (CA4). Other interviewees suggested measuring impact pathways that involved evaluating ineffective service models (“I think one of the whole objectives of the TAAHC is to be able to tell the HHSs at the end of the day whether all their activity appears to have had an impact on the people”, CA1); access to data to enable evaluation (“It's about knowing that we've made a difference and improved things”, HSE8); evidence‐informed “policies and procedures” in the health system (HSE6); and research projects that bridge translation gaps between discovery and testing in clinical practice (“[Success is the number of] projects that have been successfully completed and gone on to the implementation stage”, CA1).

##### Health impacts

Despite a widely held perception that TAAHC is ultimately aimed at improving health outcomes, very few interviewees suggested metrics at this level. One interviewee described the challenges inherent in measuring health outcomes attributable to research:

“I'd say that success means that the person in Camooweal [a remote inland town] is getting a better deal than they are now; however you measure that! And that's a research question on its own.” ( UE2)

Another interviewee suggested the use of epidemiological data to track population health impacts:

“I think we should also be looking at population health as an ‘overall’ in the areas that we're targeting as a priority. So doing some more epidemiological studies, looking at what kind of an impact it has had.” ( CA2)

##### Broader economic and social impacts

The CAHS framework separates economic and social impacts into activity, commercialisation, wellbeing, socio‐economic benefits, and health benefit per health care dollar. The one interviewee who addressed this category suggested that indicators of TAAHC's success should include financial returns to the health system:

“I would expect that you get at least a two‐fold return on the investment, measured, quantifiable in real terms, and that can be in terms of productivity or efficiency dividends, and that might relate to improved models of clinical care or ways of practice or delivery mechanisms or ways of doing business.” ( HSE12)

### 4.2 Key challenges

Despite the high aspirations and expectations, multiple experienced and anticipated challenges to achieving TAAHC aims were identified by interviewees, and these were reinforced in both TAAHC documentation and by researcher observation.

#### 4.2.1 Trust and communication

Collaboration concerns largely centred on the member organisations' perceived capacity to “influence the agenda” (HSE3) of TAAHC. A degree of parochialism was perceived to exist in the northern Queensland localities and was described as manifesting in “a low level of trust between the TAAHC member organisations” (TAAHC Review Report, 2017). Many of the interviewees also described feeling themselves, or observed in others, a lack of trust between the TAAHC partners. One health system executive saw low trust levels as reflecting a perception among health system members that the “big bold universities [are] trying to take money off the health services” (HSE5). Another health system executive saw some of the collaboration challenges as personal, reflecting tensions between one or more of the key individuals involved in TAAHC (HSE6). Nonetheless, there was widespread agreement that overcoming such impediments was necessary to achieving success within TAAHC. One health system executive described a need for “a level of reciprocity in the relationship and mutual benefit”, warning that “if TAAHC can't work that out, then I don't think anything will happen” (HSE4).

The researchers also observed challenges in the nature of communication structures, owing to the vast distances between the organisations and the need for some meetings to be held by video‐conference or tele‐conference to save on travel costs and time; challenges also described in the TAAHC Review Report (2017). Meeting minutes also indicated attempts by the TAAHC Steering Committee to develop more reliable web‐based meeting platforms (2015). Frequent turnover of executive‐level personnel in the partnering organisations was also seen as a challenge to building relationships and sustaining the collaboration: one interviewee expressed frustration at having to “start all over again” when a “major champion and supporter” leaves the region (UE2). Another questioned whether TAAHC “momentum [could] continue when different people come in and out” at the leadership levels in the health services (HSE10).

#### 4.2.2 Understanding value and return on investment

A challenge described by nearly all health system executives was a sense that either they or their health system colleagues were having difficulties understanding TAAHC's role and purpose, which affected their perception of its value and subsequent motivation to invest in its translational research agenda. Two interviewees—a health system executive (HSE1) and a clinical academic (CA3)—described the TAAHC initiative as “nebulous” and difficult to sell. There was also a sense among some interviewees that a prolonged focus on setting up the governance framework for TAAHC had detracted from the sort of activity that might have increased understanding and perceptions of value of TAAHC, such that “we seem to be a little bit stuck on setting ourselves up and how it would work rather than the actuality of what it might do.” (HSE9).

While interviewees generally saw building translational research capacity as positive, some were uncertain about what the “tangible” returns on investment would be (HSE1), and in what timeframes any benefits would become apparent. TAAHC's research agenda was broadly understood as the most significant expense associated with the initiative, with one university executive observing that “somebody has to pay for it” given that “research funding agencies don't want to pay for it in its entirety” (UE4). One document described the costs as including salaries of research teams as well as access to analytical and research design expertise such as in biostatistics and health economics (TAAHC Research Development Report, 2016). A university executive also saw “small pots of money” as necessary to “incentivise that sort of agility that encourages people to go out and innovate and test out [their ideas]” (UE3). Concerns about the establishment and recurrent costs of TAAHC were seen to have persisted for some years, and some interviewees reflected that multiple meetings of the TAAHC Steering Committee had been focussed on this issue, with one observing an understanding that “if you can't pay, you can't play” (HSE9). This was described as a particular challenge for the smaller parties to TAAHC; one health system executive described their “much smaller budget” prohibiting “the sort of funds that TAAHC would like to have committed.” (HSE6)

#### 4.2.3 Health system receptiveness to building a research culture

Two interviewees suggested that the health services' concerns about the costs of participating in TAAHC reflected a “cost containment” culture wherein the true and sometimes “intangible” benefits of research were not fully understood or valued (UE4, HSE5). One saw “innovative thinking at the coalface” in health services as requiring an “entrepreneurial culture” involving researcher autonomy (UE4); however, a clinical academic described the current culture in the state's health bureaucracy as “very anti‐research” (CA4). A university executive similarly reflected that “‘academic’ is a bit of a dirty word” in the region's health services, with “clinicians highly suspicious of academics” due to a history of limited positive interaction between the two cultures (UE3). The different organisational cultures were apparent in interviewees' conflicting attitudes towards research timeframes: one university executive stressed the need for long timeframes to deliver “research projects of substance”:

“You can't do it in five years and say this is what success is. It's going to take a while.” ( UE3)

In contrast, a health system executive described the challenges to policy‐makers of not only the long timeframes associated with research but also the specific nature of research findings:

“[One challenge with] the academic world is that it's academic, true academia … it can't help me. Because I've got to deal with today's money, by 30 June. And then some of the [research] that's done might be close [to what we need] but it might not be quite right either.” ( HSE7)

Health system performance indicators that overwhelmingly incentivise non‐research activity were described by a university executive as a structural impediment to growing the TAAHC research effort (UE1). A clinical academic also described a “risk averse” culture within both academic and health service TAAHC members which had led to excessive “red tape” in research approvals processes for potentially impactful projects (CA4). Another direct challenge was described as the high turnover of health workforce at both management and clinician levels, which was seen to hinder health system attempts to grow a research‐capable workforce and enabling culture.

#### 4.2.4 Prioritising and influencing the research agenda

Some uncertainty was expressed by interviewees around whose interests would be reflected in the outputs of TAAHC and its downstream benefits. Some saw a risk that TAAHC's research agenda could be directed away from population‐focused aims by the self‐interest of either individual organisations (CA4) or individual people (CA3; HSE11); either for reasons of prestige, money, or personal ambition. One clinical academic based in a health service expressed a concern that research in universities tends to be driven by what gets funded rather than population “burden of disease”, and this could diminish TAAHC's capacity to prioritise:

“I don't think we necessarily have confidence that a closer association with the university is going to fix [the health issues of importance in the interviewee's health service] … because they tend to focus on things that are sexy and generate funding for research … there are some topics that are very easy to get funding for.” ( CA4)

Multiple interviewees expressed a preference for TAAHC's research agenda to prioritise research addressing “the lived experience of the communities of north Queensland” (HSE6) over “blue sky biomedical” (CA3) or “laboratory quantitative” (HSE6) research. At a national level, a university executive critiqued historically “elitist” research and research funding paradigms in Australia and observed a risk that “while [research] remains a self‐regarding industry” it may continue to reward “arcane” research topics with little “social value” (UE4). A non‐clinical academic similarly saw current health and medical research paradigms disproportionately rewarding “Western” approaches to research which included “finding new solutions and new treatments” when research agenda‐setting should instead draw on “community participation” and focus on “community benefit” (NCA1). Another interviewee described a potential future risk that the “metropolitan” AHCs in Australia might disproportionately influence the national AHC research agenda (UE2).

#### 4.2.5 Structure of the health system

The different federal and state jurisdictional responsibilities for health care were seen by health system interviewees as a more general barrier to efforts to improve the operation of the region's health system, which was relevant to TAAHC's ultimate population‐focused aim. One health system executive described the challenge of delivering health care when “we have these different buckets of money that we can only spend on certain things” (HSE8). Another health system executive from an HHS in TAAHC elaborated on the challenges of meeting population health priorities when they only had a mandate to deliver certain types of services:

“Primary health care is really becoming more and more important … [but] we're not funded to do most of the primary health care service delivery...Under the service agreement, we can only do what we're funded to do, because if we fund more it's got to be from our own funds, and there's a limit to how much we can [fund that way].” ( HSE4)

One clinical academic reflected on the constant changes to health system structures, which were seen to hinder attempts to build and sustain services: “just as they're starting to stabilise and make progress, it gets changed again” (CA4).

## 5 DISCUSSION

This paper reports the results of a qualitative case study of a regional Australian AHC, called the TAAHC, which sought to examine the intended impact pathways and beneficiaries of TAAHC and to identify the experienced and anticipated challenges in achieving these aspirations. This paper presents one of the first empirical studies to explore the establishment of AHCs in Australia and contributes to an identified need for empirical research on the barriers to the successful establishment of AHC structures in Australia and possible determinants of their success.^2^

The aims of TAAHC reflect expectations of the contributions of AHC models in Australia which conceptualise AHCs as vehicles for improving health through enhancing translational research capacity, and which also see collaborative arrangements that include both health service delivery and academic organisations as imperative to such enhancement.^3^, ^5^ TAAHC's focus on health service improvement, education, and research translation also reflects international mission‐based definitions of AHCs.^1^ Beyond the tripartite mission, we identified additional aspirations in TAAHC relating to increasing prosperity, building life sciences industries and financial returns on investment, reflecting a similar focus among AHCs in the United Kingdom which were created to serve both a health improvement and a “wealth” agenda.^27^

**Figure 4:**
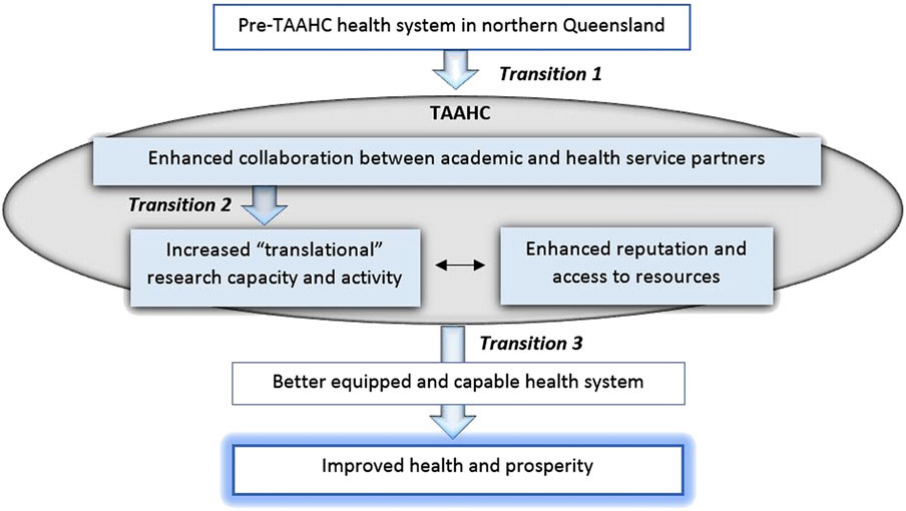
Intended impacts of TAAHC showing three hypothetical transitional stages from establishment. Shaded boxes indicate the key aims of TAAHC, with large arrows representing the main transitions described in the data

Reflecting our analysis of TAAHC aspirations, Figure 4 presents the main intended impact pathway of TAAHC which involves a series of hypothetical and sequential “transitions” between key aims. Transition 1 represents establishing effective inter‐institutional collaboration, transition 2 represents increasing translational research capacity and activity through the collaboration, and transition 3 represents improving health, wellbeing, and prosperity for identified populations. The pathway commences with enhanced collaboration between the member organisations in TAAHC, which increases translational research capacity, leading to improved health system performance and ultimately improvements to health for defined populations.

Transition 1, representing establishing effective inter‐institutional collaboration between the component organisations in TAAHC, reflects the TAAHC “whole is greater” aim and links to the CAHS “building capacity” category. Key stakeholders in TAAHC identified *trust and communication* to be a challenge within this transition associated with the TAAHC establishment experience. This challenge reflects similar difficulties encountered elsewhere in bringing together multiple organisations with different accountability structures around the combined research, service and education missions.^17^, ^28^, ^29^, ^30^ TAAHC's vast, rural, and distributed geography appeared to amplify these challenges, pointing to the relevance of geographic context, and associated governance and communication structures and processes, in influencing capacity for relationship building between participating organisations in AHCs.

Transition 2 represents the pathway towards the key aim of TAAHC to build translational research capacity and activity through the collaborative model of TAAHC, which also links to the CAHS “building capacity” category. Findings demonstrated both experienced and anticipated challenges to realising this transition; these included *understanding value and return on investment* and *health system receptiveness to building a research culture* and reflect challenges documented elsewhere in literature on research capacity building in health care settings. For example, different ideas among TAAHC stakeholders about the role of research and its potential contributions are addressed in literature on the cultural disconnect between academic and policy worlds^31^ and on the role of co‐production in health care settings.^32^ Some of the issues appeared beyond the capacity of the TAAHC partners themselves to address. For instance, perceived deficiencies in Australia's health and medical research funding systems included incomplete resourcing of the research endeavour and apparent urban bias; these were identified as current and potential barriers to generating sufficient resources to support the research aspirations of TAAHC but are national policy issues. Reinforcing the apparent complexity of this transition, some issues also appeared to be linked with TAAHC aspirations in a way that was mutually recursive; for example, TAAHC aims to improve capacity to attract and retain talented workforce, but doing so requires an existing cadre of informed and motivated health system leaders.

Transition 3 represents the pathway towards improving health and involves improving the functioning of the region's health system. This transition was effected mostly by increased translational research capacity (“research that has impact”) but also directly by enhanced collaboration (“whole is greater”) and reputation and resources (“voice of the north” and “attract clinical experts”). As TAAHC was still undergoing establishment at the time of data collection, the challenges we identify as relevant to this transition—*prioritising and influencing the research agenda* and the *structure of the health system*—reflected assumptions about TAAHC's role rather than experience. A substantial body of literature addressing the research‐to‐health benefit pathway of transition 3 includes that on “knowledge mobilisation” within health care settings,^33^ but there are notable gaps. For example, while an association has been identified between research‐active health systems and improvements in patient outcomes,^34^ the causal pathways remain unclear.^30^

Perhaps reflecting TAAHC's population health orientation, we identified a preference among key stakeholders for TAAHC outputs to effect systems change and improvement, rather than deliver the sorts of “discovery‐oriented” research products traditionally associated with AHCs elsewhere.^35^ Based on this finding, and on global interest in the equity role of AHCs,^12^ we suggest that future research on knowledge‐to‐action pathways include broader populations (not only individual patients) as intended beneficiaries of AHCs. We also identified a distinction between the scope of TAAHC's intended activity and that of the member organisations; in that TAAHC was seen as a vehicle for the collaboration, translational research and reputation and resources agendas, whereas responsibility for delivering the ultimate population and societal impacts lies with the (ostensibly better equipped and capable) health service partners themselves. This distinction may reflect the unlinked partners model of AHCs in Australia as compared with, for example, more integrated academic‐clinical service relationships observed in the United States.^19^

## 6 STRENGTHS AND LIMITATIONS

We highlight some potential limitations of the study. First, as the study is a single case study, the findings are likely to be context specific with conditions such as geography, health system structures, establishment stage, and governance structures potentially influencing transferability. To address this, further cross‐case research is currently being undertaken by the researchers to improve analytic generalisability of the findings to other AHCs in Australia and overseas. Second, we recognise potential omission of particular interviewee perspectives; a risk that we sought to minimise through using multiple and diverse data sources identified through both purposive and theoretical sampling methods. We highlight other methodological strengths of the study such as data triangulation, member checking, and reflexive practice which collectively improve trustworthiness of the findings. We also followed the COREQ checklist^36^ to strengthen quality of reporting.

## 7 CONCLUSION

Our case study of a regional Australian AHC contributes to the empirical evidence base on the role of AHCs internationally and has policy relevance for ongoing initiatives to establish and resource AHC models in Australia. Three interdependent transitions from enhancing collaboration through to improving population health—forming the main impact pathway in TAAHC—were identified in the study along with key challenges at each transitional stage. TAAHC aims reflected national expectations of AHCs suggesting potential for the impact categories used in the study to be combined with assessment of aspiration to inform future work to evaluate the activity and contributions of AHCs in Australia. The identified challenges, particularly those reflecting the establishment experience of TAAHC, may also be relevant to other emerging AHCs. Future research should seek to strengthen the evidence base supporting each intended transition; this should include broader populations as intended beneficiaries and also consider the entire system of transitions in order to capture possible interdependencies.

## CONFLICT OF INTEREST

Three of the authors are employed by James Cook University, which is an organisational partner of the Tropical Australian Academic Health Centre. The lead author also previously held an operational role within the Centre. No other conflicts are identified.

## FUNDING AND APPROVALS

Ethics approval was received from Townsville Hospital and Health Service Human Research Ethics Committee on 12 July 2017, and site‐specific approvals were also received from each of the five participating Hospital and Health Service locations. Reciprocal approval was also provided by the James Cook University Human Research Ethics Committee on 7 September 2017. The lead author is supported by a postgraduate scholarship from James Cook University, and Dr Ovseiko is supported by the National Institute for Health Research (NIHR) Biomedical Research Centre, Oxford.
